# Serum proteases prevent bacterial biofilm formation: role of kallikrein and plasmin

**DOI:** 10.1080/21505594.2021.2003115

**Published:** 2021-12-14

**Authors:** Jesús Arenas, Zalan Szabo, Jelle van der Wal, Coen Maas, Tahira Riaz, Tone Tønjum, Jan Tommassen

**Affiliations:** a Department of Molecular Microbiology and Institute of Biomembranes, Utrecht University, Utrecht, The Netherlands; b Unit of Microbiology and Immunology, Faculty of Veterinary, University of Zaragoza, Zaragoza, Spain; c Research and Development Department, U-Protein Express BV, Utrecht, The Netherlands; d Department of Clinical Chemistry and Haematology, University Medical Center Utrecht, Utrecht, The Netherlands; e Department of Microbiology, University of Oslo, Oslo, Norway

**Keywords:** *Neisseria meningitidis*, *Bordetella pertussis*, biofilms, serum proteases, NHBA, IgA protease, FHA, NaLP, eDNA, kallikrein, plasmin

## Abstract

Biofilm formation is a general strategy for bacterial pathogens to withstand host defense mechanisms. In this study, we found that serum proteases inhibit biofilm formation by *Neisseria meningitidis, Neisseria gonorrhoeae, Haemophilus influenzae*, and *Bordetella pertussis*. Confocal laser-scanning microscopy analysis revealed that these proteins reduce the biomass and alter the architecture of meningococcal biofilms. To understand the underlying mechanism, the serum was fractionated through size-exclusion chromatography and anion-exchange chromatography, and the composition of the fractions that retained anti-biofilm activity against *N. meningitidis* was analyzed by intensity-based absolute quantification mass spectrometry. Among the identified serum proteins, plasma kallikrein (PKLK), FXIIa, and plasmin were found to cleave neisserial heparin-binding antigen and the α-peptide of IgA protease on the meningococcal cell surface, resulting in the release of positively charged polypeptides implicated in biofilm formation by binding extracellular DNA. Further experiments also revealed that plasmin and PKLK inhibited biofilm formation of *B. pertussis* by cleaving filamentous hemagglutinin. We conclude that the proteolytic activity of serum proteases toward bacterial adhesins involved in biofilm formation could constitute a defense mechanism for the clearance of pathogens.

## Introduction

Biofilm formation is a strategy commonly used by pathogenic bacteria to resist the host immune system. In fact, biofilms are the origin of multiple chronic and recurrent infections that are difficult or impossible to treat [1]. Biofilms contribute to nearly 80% of recalcitrant hospital infections and result in high mortality and morbidity [2] as well as in tolerance and resistance to antibiotics through diverse mechanisms [3]. Also, many opportunistic microorganisms use biofilm formation to colonize the host’s mucosal surfaces; thus, they resist mechanical forces derived from mucus flow, coughing, or sneezing. Under particular conditions, these microorganisms can get access to the blood stream causing systemic infections that can compromise the life of the host. In the blood stream, the bacteria can adhere to endothelial cells in capillaries and form microcolonies. Microcolonies are associations of microorganisms, often derived from one clone, surrounded by an extracellular matrix; they are the basis of biofilm formation. Microcolonies are resistant to shear forces generated by blood flow [4,5]. They help to resist immune defenses and affect the barrier function of the endothelium [6], which may facilitate the bacteria to pass the blood–brain barrier. Understanding the host response to microcolony and biofilm formation will open novel avenues to improve outcomes for many pathogenic infections.

*Neisseria meningitidis* is a Gram-negative bacterium that usually lives as a commensal in the nasopharynx of humans without causing any symptoms. Around 10–30% of the population is colonized by the bacterium, but this rate can be higher in adolescents and young adults [7]. Once the bacteria enter the host, they adhere to the epithelial surfaces of the nasopharynx-forming microcolonies [8]. *N. meningitidis* is also the etiological agent of the meningococcal disease when it reaches the blood stream and the cerebrospinal fluid after crossing epithelial and endothelial barriers [9].

*N. meningitidis* can produce well-organized biofilms, which vary in architecture between strains [10]. Several factors are involved in the formation of these biofilms by mediating interbacterial and bacterium–substratum interactions. A prevalent component in these interactions is extracellular DNA (eDNA), which forms part of the extracellular matrix [11]. eDNA is essential to initiate biofilm formation in a large variety of strains of different clonal complexes. However, strains of clonal complexes 8 and 11 can initiate biofilm formation independent of eDNA [11]. Several proteins act to anchor eDNA at the bacterial surface, such as the neisserial heparin-binding antigen (NHBA), the α-peptide of IgA protease (IgAp) [12], and the autotransporters AutA [13] and AutB [14], all which are important virulence factors [15]. While AutA and AutB are rarely expressed in *N. meningitidis* [13,14], NHBA and IgAp are produced in the vast majority of the strains and, therefore, they have a critical role in biofilm formation.

**Figure 1.**
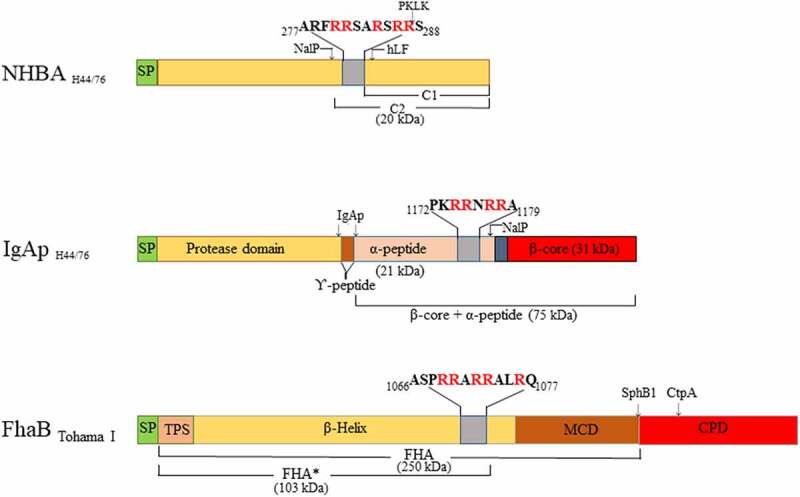
**Overview of the domains of neisserial heparin-binding antigen (NHBA), neisserial IgA protease (IgAp), and the *B. pertussis* filamentous hemagglutinin precursor FhaB** (not drawn to scale). Sequence data refer to the proteins of *N. meningitidis* strain H44/76 and *B. pertussis* strain Tohama I. The domains and subdomains are differently colored. Approximate positions for processing sites are indicated with arrows, and the molecular mass of the resulting fragments is shown. The position and the sequences of the Arg-rich regions are depicted. hLF, human lactoferrin; PKLK, plasma kallikrein; TPS, two-partner secretion domain; MCD, mature C-terminal domain; CPD, carboxy proximal domain; SP, signal peptide

NHBA is a lipoprotein and a component of the Bexsero vaccine commercialized to prevent meningococcal disease [16]. It contains a C-terminal domain composed of an eight-stranded β-barrel and an unstructured N-terminal domain [17], separated by an arginine-rich region that binds heparin [18], heparan sulfate [19], and eDNA [12] (Figure 1). NHBA is critical for biofilm formation by virtue of its eDNA-binding ability. NHBA can be cleaved immediately upstream of the arginine-rich region by the autotransporter protease NalP [18] (Figure 1), whereby the C-terminal segment including the positively charged region is released into the milieu, where it affects endothelial integrity [20]. However, NalP does not cleave all NHBA molecules, and the uncleaved proteins that remains at the cell surface suffice for biofilm formation [12]. Expression of *nalP* is prone to phase variation because of the presence of a variable number of nucleotide repeats located immediately after the start codon [21]. Thus, when *nalP* is not expressed, all NHBA molecules are retained at the cell surface, which enhances eDNA binding and, consequently, biofilm formation [12]. Additionally, NalP processes IgAp [22]. Like NalP, IgAp is an autotransporter. Autotransporters are composed of a translocator domain (TD), located at the C-terminal end and fully integrated in the outer membrane, and a secreted passenger domain (Figure 1). The passenger domain of IgAp is composed of a protease domain, a linker, and an α-peptide (Figure 1). The protease domain is released from the cell surface by an autocatalytic mechanism, while the protease domain together with the α-peptide and the linker is released by the protease activity of NalP (Figure 1). The α-peptide contains one to four arginine-rich segments, while NHBA contains one. These segments bind eDNA and contribute to biofilm formation when *nalP* is not expressed [12]. In contrast to NHBA, all α-peptide molecules are released from the cell surface by NalP, and, thus, this domain does not contribute to biofilm formation when NalP is produced, at least not in the model strain HB-1.

As for *N. meningitidis*, eDNA is a relevant constituent of the biofilm matrix of several other human pathogens that habit the human mucosa. Examples are non-typeable *Haemophilus influenzae* (NTHi), *Staphylococcus aureus*, and *Bordetella pertussis*. NTHi is a commensal bacterium that lives in the upper and lower respiratory tract and can cause sinusitis, acute otitis media, conjunctivitis, and bacteraemia. NTHi produces extracellular polymeric substances to promote biofilm formation, including eDNA , which has been found to be associated with pili, and several adhesins [23]. *S. aureus* colonizes the nasal mucosa, and, after epithelial breach, can disseminate to other areas of the body and reach the blood system. The extracellular matrix of *S. aureus* biofilms is mainly constituted by a polysaccharide named polysaccharide intercellular adhesin (PIA) [24]. However, staphylococcal biofilms can also be formed in a PIA-independent manner, whereas protein A, cell-wall-anchored proteins, such as fibronecting-binding proteins FnBPa and FnBPB, and the biofilm associated protein Bap promote the biofilm formation of biofilms with amainly proteinaceous matrix [25]. Besides, staphylococci can also generate eDNA-dependent biofilms. eDNA is a component of the matrix that contributes to the initial surface attachment. The importance of eDNA for this bacterium is highlighted by the large variety of mechanisms used to release DNA [26]. Cytoplasmic proteins associated with the cell surface function as eDNA-binding proteins by establishing electrostatic interactions with eDNA [27]. Finally, *B. pertussis* is a gram-negative bacterium that colonizes the respiratory tract and causes pertussis disease, characterized by a succession of paroxysmal forceful coughs and energetic inspiration attemps. *B. pertussis* produces several adhesins such as filamentous hemagglutinin (FHA), pertactin, fimbriae, and BrkA, which are involved in biofilm formation [28]. FHA is a large protein that promotes the structural integrity of the biofilm by mediating cell-substratum and interbacterial interactions [29]. The biofilm matrix is composed of several extracellular polymeric substances including polysaccharides, proteins, metabolites, and eDNA, the composition of which is strain dependent [28]. FHA contains arginine-rich regions that were proposed to bind eDNA [10]. Together, a multitude of evidence indicates that eDNA is a common component of the biofilm matrix of many pathogens that associates with particular cell surface-exposed proteins or exoproteins, probably to strengthen cell–matrix interactions.

In this study, we found that foetal calf serum (FCS), which is commonly used as a reagent in various assays, inhibits the formation of biofilms by many human pathogens, suggesting the existence of an innate defense mechanism in blood against biofilm formation. To identify the responsible molecules, we used *N. meningitidis* as the model organism and evaluated the integrity of molecules critical for biofilm formation in this organism. We succeeded to identify serum proteins responsible for inhibiting biofilm formation and established the mode of action.

## Material and methods

### Proteins, protease inhibitors, and antisera

Recombinant NHBA and α-peptide of IgAp of H44/76, both containing an N-terminal His-tag, were produced and purified from *Escherichia coli* BL21(DE3) as previously reported [12]. Plasmids pET-H-IgApAP and pET-H-NhbA (Table 1) were used to transform *E. coli*. The recombinant NHBA consisted of an N-terminal His tag followed by 385 amino-acid residues (residues 35–420) of NHBA of strain H44/76. The recombinant α-peptide of IgAp also contained an N-terminal His tag followed by 177 amino-acid residues (residues 1005–1182) of IgAp of strain H44/76. Plasma kallikrein (PKLK), α-coagulation Factor XII (FXII)a and β-FXIIa were from Enzyme Research Laboratories (South Bend, IN, USA). Streptokinase was from CSL-Behring (Breda, The Netherlands). Plasminogen was purified from citrated human plasma as previously described [30]. The protease inhibitors Kallistop and ɛ-aminocaproic acid (ɛACA) were from Sekisui Diagnostics (Burlington, MA, USA) and Sigma-Aldrich (St Louis, MO, USA), respectively. Antisera directed against the α-peptide and against the TD of IgAp were described previously [31]. Antiserum directed against the full lengh NHBA and monoclonal antibodies against FHA [32] were kindly provided by Dr Mariagrazia Pizza (GSK, Sienna, Italy) and Dr Françoise Jacob-Dubuisson (INSERM, Lille, France), respectively.

**Table 1.** Strains and plasmids used in this study

*item*	Relevant characteristics^1^	Source or reference
**Strains**		
*E. coli*		
BL21 (DE3)	Overexpression strain	Laboratory collection
*N. meningitidis*		
HB-1	Derivative of H44/76 (B:15:P1.7,16) of cc32 with the capsule locus disrupted. Ery^R^	[59]
HB-1 Δ*nalP*	HB-1 with *nalP* deleted. Ery^R^, Kan^R^	[22]
HB-1 Δ*nalP*_GFP	HB-1 Δ*nalP* with insertion of *opaBP_H_-gfp* into *hrtA* locus. Ery^R^, Kan^R^, Rif^R^	[34]
HB-1 Δ*nhbA*	HB-1 with *nhbA* deleted. Ery^R^, Kan^R^	[12]
BB-1	Derivative of B16B6 (B:2a:P1.2) of cc11 with the capsule locus replaced by Ery^R^	[12]
*N. gonorrhoeae*		
FA1090	ATCC 700825	Reference strain
VG3		Laboratory collection
*H. influenzae*		
R2866	Non-typeable *H. influenzae*	[60]
*S. aureus*		
NCTC 8178	ATCC 13420	[61]
*B. pertussis*		
B213	Str^R^ derivative of strain Tohama I, Nal^R^	[62]
**Plasmids**		
pETx507_AutAp	pET16b plasmid containing sequences for a polyhistidine-tag and TEV recognition sites behind the sequence of the passenger domain of *autA*	Laboratory collection
pET-H-IgApAP	pET16b derivative encoding α-peptide of IgAp	[12]
pET-H-NhbA	pET16b derivative encoding recombinant NHBA	[12]

^1^Ery^R^, erythromycin resistant; Kan^R^, kanamycin resistant; Rif^R,^ rifampicin resistant; Nal^R^, nalidixic-acid resistant; Str^R^, streptomycin resistant.

### Bacterial strains and growth conditions

Bacterial strains used in this study are listed in Table 1. *N. meningitidis* and *N. gonorrhoeae* strains were grown at 37°C on GC agar (Oxoid, Basingstoke, United Kingdom) supplemented with Vitox (Oxoid) in a candle jar. Liquid cultures were grown in tryptic soy broth (TSB) (Oxoid) or, where indicated, in RPMI 1640 medium (Gibco-BRL) supplemented or not with either FCS (PAA Laboratories, Pasching, Austria) or human serum (Sigma-Aldrich) at 37°C with constant shaking at 110 rpm. *S. aureus* was grown on tryptic soy agar (Oxoid) and in TSB supplemented or not with FCS at 37°C. *H. influenzae* was grown on brain heart infusion (BHI; Panreac, Catellar del Vallés, Spain) agar plates supplemented with 1 µg/ml of hemin (Sigma-Aldrich) and 2 µg/ml of β-nicotinamide adenine dinucleotide (Merck) at 37°C in a candle jar. For liquid cultures, the bacteria were grown in BHI supplemented as described above while shaking at 150 rpm at 37°C. *B. pertussis* was grown on Bordet-Gengou (BG; BD LifeSciences) agar supplemented with 15% defibrinated sheep blood (Biotrading, Mijdrecht, The Netherlands) or as cultures in Verwey medium [33] at 35°C with shaking at 175 rpm. *E. coli* strains were grown in lysogeny broth supplemented with 100 µg/ml of ampicillin.

To test the effect of FCS on bacterial viability, cultures of *N. meningitidis* strain HB-1 in TSB medium were adjusted to an optical density at 550 nm (OD_550_) of 1.0, and FCS was added to a final concentration of 5%. After 1 h incubation in 24-wells plates, samples were serially diluted and plated on GC plates, and colony-forming units (CFU) were counted after overnight growth.

### Biofilm formation assays

Biofilms were formed under static conditions in 24- or 96-wells plates as previously described [12–14] with modifications. Cultures of *N. meningitidis, N. gonorrhoeae, S. aureus*, or *H. influenzae* grown for 5 h, or of *B. pertussis* grown for 48 h, were adjusted to an OD_590_ of 1, and 500- or 100-µl samples were seeded per well in 24- or 96-wells plates, respectively. Where indicated, FCS, plasmin, PKLK, α-FXIIa, or β-FXIIa was added to each well at different concentrations simultaneously with the bacterial cultures. Plasmin was produced by incubating 200 µg/ml of plasminogen with 20 U/ml of streptokinase for 20 min at 37°C before use. Where indicated, proteases or FCS were incubated with 50 nM Kallistop or 200 mM ɛACA for 1 h at 37°C before incubation with the bacteria. Bacteria were incubated together with the supplements for biofilm formation for different time periods. *N. meningitidis, N. gonorrhoeae, H. influenza, S. aureus,* and *B. pertussis* were incubated during 1, 3, 8, or 12 h as indicated. Then, the medium was removed from each well, and the biofilm was washed twice with deionized water and stained with crystal violet as described [12–14]. To determine DNase sensitivity of biofilm formation, 100 µg/ml of DNase I (Sigma) was added to the cultures [12]. To determine the presence of biofilm-inhibiting activity in serum fractions recovered from chromatography columns, 50 µl of each fraction were added to 100 μl of bacterial cultures. For these assays, FCS (5% final concentration) or Tris buffer (20 mM Tris-HCl, pH 8.0) was added as positive and negative controls, respectively.

### Microscopy

For microscopy, 1- or 12-h-old biofilms were formed under static conditions in 24-wells plates on glass cover slips according to [34] in the presence or absence of 1% FCS. Microscopic observations and image acquisitions were performed using a Zeiss LSM 700 confocal laser scanning microscope (Carl Zeiss) equipped with 40x/1.30 Plan-Neofluar or 63x/1.40 Plan-Apochromat oil objectives. Biofilm phenotypes were considered when observed in three independent experiments performed in duplicate. For the analysis of the structural parameters of the biofilm (biomass, average thickness, and roughness coefficient), image stacks at 0.4-µm z-intervals were acquired and analyzed with the program COMSTAT [35] in the image processing environment ImageJ (https://imagej.nih.gov/ij/).

### Fractionation of FCS

FCS was first fractionated by size-exclusion chromatography (SEC). For this, 1 ml of FCS was loaded on a 150-ml Superdex 200 Prep grade column (Pharmacia Biotech) using an AKTA Prime Plus system (GE Healthcare) with sterile phosphate-buffered saline (PBS) (Lonza) as the mobile phase. Eluting proteins were collected in 2.5-ml fractions, which were then analyzed by SDS-PAGE and tested for biofilm-inhibiting activity as described earlier. SEC fraction 10 was further fractionated by anion-exchange chromatography (AEC). For this, the buffer of the fraction was exchanged for sterile Tris buffer10 (10 mM Tris-HCl, pH 8.0) using a HiPrep 5-ml desalting column (GE Healthcare). The desalted sample was then loaded onto a 1-ml Resource Q column (Pharmacia) equilibrated in Tris buffer10. The column was washed with 10 column volumes of Tris buffer10, and bound proteins were eluted with a gradient of sterile 0–500 mM NaCl in Tris buffer10 in ten column volumes. Along the salt gradient, 0.3-ml fractions were collected and analyzed on a 4–12% NuPAGE Bis-Tris gel (ThermoFisher Scientific), which was then stained with Page Blue protein staining solution (ThermoFisher Scientific).

### Cleavage of NHBA, α-peptide, and FHA by serum proteases

Cells of *N. meningitidis* HB-1 and HB-1∆*nalP* were adjusted to an OD_550_ of 1.0 in TSB medium and incubated with fractions obtained from SEC or AEC. As positive and negative controls, bacteria were incubated with 1% FCS or TSB, respectively. After 1 h incubation at 37°C, bacterial cells were collected by centrifugation for 10 min at 4000 rpm in a table centrifuge, and the pellet fraction was analyzed by SDS-PAGE and Western blotting. To analyze the specific contribution of serum proteases, 0.5 µg of recombinant NHBA, cell envelope preparations of HB-1 Δ*nalP*, which were prepared as in Arenas et al. [13], or whole cells of *B. pertussis,*which were scraped from plates after 24 h of growth, adjusted to an OD_600_ of 1.0 and washed with PBS, were incubated with different concentrations of plasmin, PKLK, α-FXIIa and β-FXIIa for 1 h at 37°C in TSB medium. Plasmin was generated from plasminogen with streptokinase as described above. The samples were prepared for SDS-PAGE as described below .

### SDS-PAGE and Western blotting

Before SDS-PAGE, samples were diluted 1:1 in double-strength sample buffer and heated for 10 min at 100°C. SDS-PAGE was performed on 14%, 12% or 8% polyacrylamide gels in a discontinuous buffer system. Proteins separated on gels were stained with Coomassie brilliant blue G250 or transferred to nitrocellulose membranes. These membranes were then blocked with PBS supplemented with 0.1% (v/v) Tween 20 and 1% (w/v) nonfat dried milk (PBS-T-M). Next, the membranes were incubated with the primary antisera, and subsequently with horseradish peroxidase-conjugated goat anti-rabbit IgG or anti-mouse IgG antibodies (Biosource International). All incubations were performed for 1 h and followed by three washes for 15 min with PBS-T-M. Blots were developed with the Pierce ECL Western blotting substrate. The luminescence on the blot was detected with a bioimaging system (Bio-Rad), and the apparent molecular weight of the bands of interest was assigned using Image Lab software.

### Far dot-blotting assay

To detect binding of proteins to DNA, plasmid pETx507_AutAp (Table 1) was used as target DNA. The plasmid was first digested with NotI for 1 h at 37°C. After heat inactivation of the enzyme (20 min at 85°C), 1 µl of plasmid at different concentrations and, as a control, 1000 ng of bovine serum albumin (BSA, Sigma-Aldrich) was deposited on a positively charged Hybond-N^+^ nylon membrane (Amersham/GE Healthcare, Chicago, USA). After 5 min at room temperature, the DNA was cross-linked to the membrane by exposure to UV at 312 nm during 10 min. The membranes were then blocked in PBS-T-M during 1 h and probed with 0.1 g/l of undigested or FCS-treated recombinant polypeptides for 16 h at 4°C with constant shaking. For FCS treatment, the recombinant polypeptides were incubated during 1 h at 37°C with 1 ml of FCS. The membranes were then washed three times with PBS-T-M, and incubated with anti-NHBA and anti-α-peptide antibodies and with secondary antibodies as described above.

### Mass spectrometry analysis

Fractions recovered from AEC were prepared for mass spectrometry analysis. Fractions 8, 13, 16, and 24 were subjected to in-solution tryptic digestion, and the purified peptides were analyzed by mass spectrometry using a data-dependent top-10 method as described in Frye et al. [36]. For peptide separation, a 146-min gradient was used with solvent A {0.1% FA/3% ACN [FA:LC-MS grade (Fluka); ACN: LC-MS grade (Merck)]} and solvent B (0.1% FA/97% ACN) using the following steps: (i) 2% solvent B from start to 5 min, (ii) 2% to 20% solvent B from 5 to 105 min, (iii) 20% to 32% solvent B from 105 to 125 min, (iv) 32% to 95% solvent B from 125 to 126 min and (v) 95% solvent B until 146 min. The raw data from the mass spectrometry analyses were further analyzed by MaxQuant software (version 1.6.0.13) against the UniProt *Bos taurus* (bovine) proteome (protein ID: UP000009136) for peptide/protein identification applying the intensity-based absolute quantification (iBAQ). The identification output file from MaxQuant with the iBAQ values was filtered using the Perseus software. MaxQuant and Perseus analyses were performed using parameters described in [37]. Common contaminants were removed, and the iBAQ values were transformed to log2.

### Statistical analysis

All data obtained for the analysis of biofilm formation were expressed relative to wild-type values before statistical analysis. Data from three independent experiments performed in duplicate were considered for statistical comparison. For the determination of changes in the biofilm characteristics, at least five randomly generated image stacks of each sample in a representative experiment, where all the strains were grown simultaneously but in different channels, were considered. Data were analyzed using an unpaired statistical *t*-test with GRAPH PAD v 6.0 (Graph Pad Software, Inc.).

## Results

### *Biofilm formation of* N. meningitidis *and other bacterial species is inhibited by serum*

**Figure 2.**
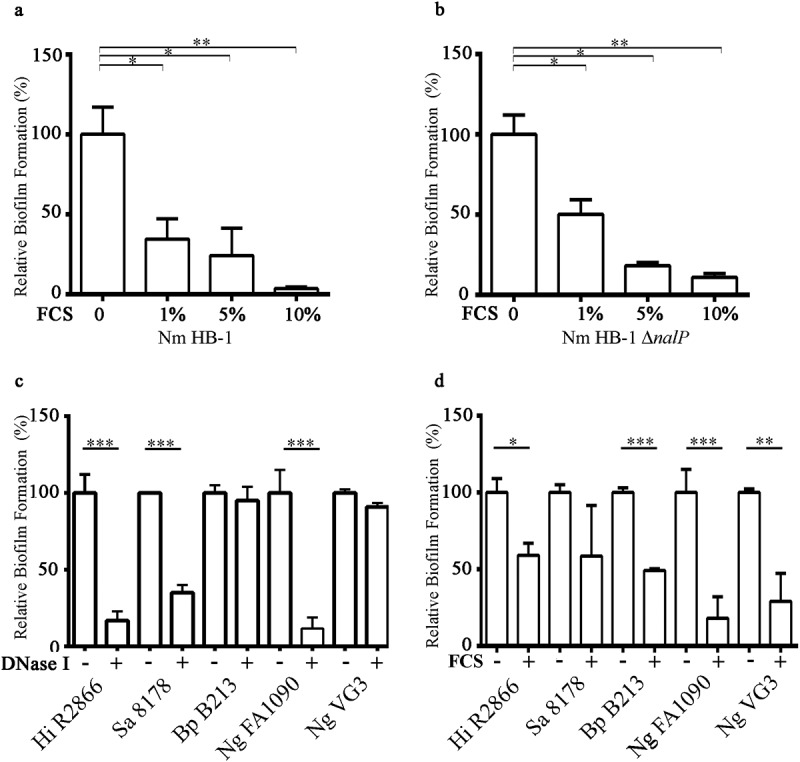
**Effect of FCS on biofilm formation**. (a, b) Impact of different concentrations of FCS in TSB on biofilm initiation of *N. meningitidis* (Nm) strains HB-1 (a) and its *nalP* mutant derivative (b). (c, d) Impact of the addition of 100 µg/ml of DNase I (c) or 10% FCS (d) in the culture medium on biofilm formation of *H. influenzae* (Hi) strain R2866, *S. aureus* (Sa) strain NCTC 8178, *B. pertussis* (Bp) strain B213, and *N. gonorrhoeae* (Ng) strains FA1090 and VG3. 1-h-old biofilms were generated for Nm, Ng 1090, Hi, and Sa, while 3-h-old and 12 h old biofilms were generated for Ng VG-3 and *Bp*B213,respectively. The data represent the means and standard deviations of three independent experiments. Values are given as relative to no treatment (without DNase I or FCS), which was set at 100. Statistically significant differences between groups were determined with unpaired *t*-test and are marked with one (*P* < 0.05), two (*P* < 0.005), or three asterisks (*P* < 0.0005)

While studying biofilm formation of *N. meningitidis* with cultured eukaryotic cell lines, we observed that the presence of 5% FCS in RPMI medium, a medium routinely used for cell culturing, drastically reduced the capacity of *N. meningitidis* strain HB-1 to form biofilms on abiotic surfaces. To investigate this phenomenon further, we first tested whether FCS also inhibits biofilm formation in TSB, a richer medium in which *N. meningitidis* forms more robust biofilms [12–14,34]. Addition of increasing concentrations of FCS to cultures of *N. meningitidis* strain HB-1 prevented the formation of 1 h old biofilms in a dose-dependent manner (Figure 2a). FCS did not affect bacterial viability (Figure S1a). Also after 12 h incubation, biofilm formation was still drastically reduced in the presence of FCS (data not shown). Similar results were obtained when human serum instead of FCS was used (Figure S1b). Thus, these experiments indicate that serum prevents initiation of biofilm formation. Biofilm initiation of *N. meningitidis* depends on the NalP expression status [12]. When NalP is not produced, positively charged polypeptides, i.e. NHBA and the α-peptide of IgAp, are retained at the bacterial cell surface where they bind eDNA, thus enhancing biofilm production. The biofilm-forming capacity of a *nalP* mutant of HB-1 was also inhibited by FCS (Figure 2b). FCS also inhibited biofilm formation of strain BB-1 to a similar extent as that of HB-1 (Figure S1b). This strain is of clonal complex 11 and, in contrast to HB-1, it uses an eDNA-independent pathway of biofilm formation and generates measurable biofilms in 8 h [12]. Thus, the effect of FCS on meningococcal biofilm formation is independent of the biofilm-forming strategy used. We also examined whether FCS inhibits biofilm formation in several other bacterial species, i.e. *H. influenzae, S. aureus, B. pertussis*, and *N. gonorrhoeae*.

The biofilm-forming capacity varied between the different strains and species. We focused on early steps of biofilm formation (i.e. biofilm initiation), following our previous observations in *N. meningitidis*. Biofilms of *S. aureus* strain NCTC 8178, *H. influenzae* strain R2866, and *N. gonorrhoeae* strain FA1090 were observed at the earliest after 1 h, while those of *N. gonorrhoeae* VG3 and *B. pertussis* B213 were detectable after 3 and 12 h, respectively. Next, we analyzed the relevance of eDNA in biofilm initiation for each species. Biofilm formation of *S. aureus* strain NCTC 8178, *H. influenzae* strain R2866, and *N. gonorrhoeae* strain FA1090 was sensitive to DNase I treatment, in contrast to that of *B. pertussis* strain B213 and *N. gonorrhoeae* strain VG3 (Figure 2c). Addition of FCS to the culture medium prevented biofilm formation of all strains and species tested, except for *S. aureus* where the effect of FCS was not statistically significant; the large inter-assay variation when FCS was added prevented statistical power. In conclusion, FCS inhibits bacterial biofilm initiation in multiple bacterial species.

### FCS alters biofilm architecture

**Figure 3.**
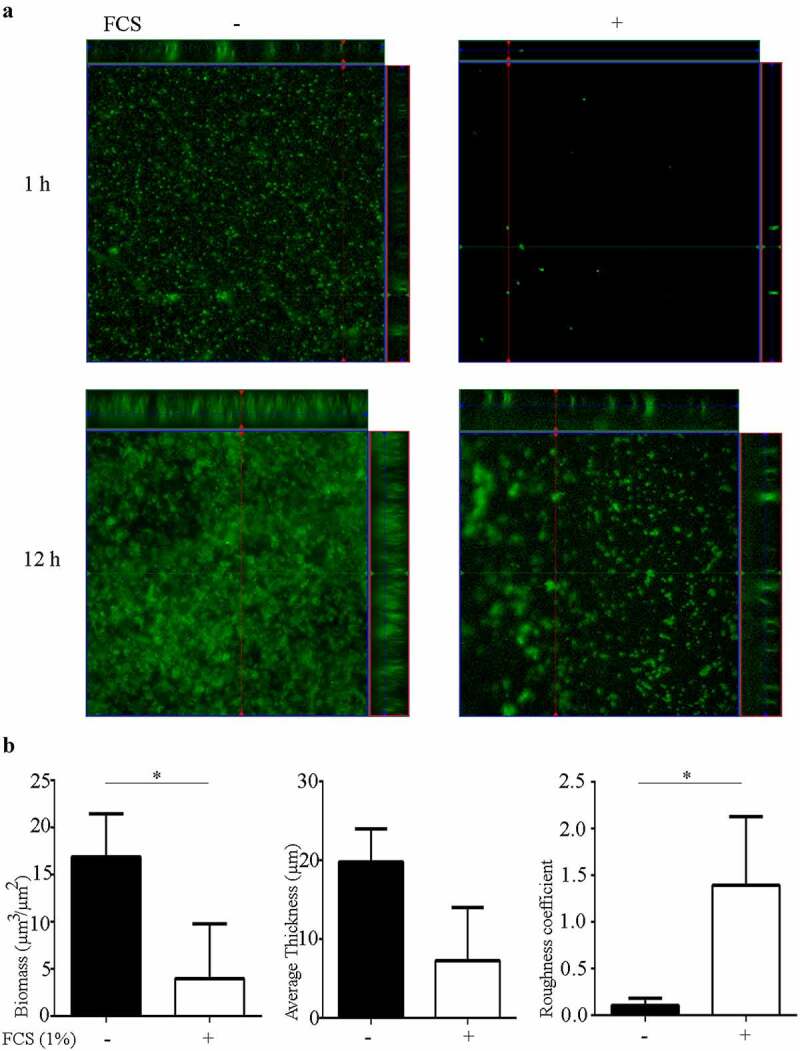
**Impact of FCS on biofilm architecture**. (a) Organization of 1- and 12-h-old biofilms of *N. meningitidis* strain HB-1 Δ*nalP*_GFP produced in TSB supplemented or not with 1% of FCS. (b) Biofilm parameters (biomass, thickness and roughness) were calculated using COMSTAT software. Values are means of data from at least five image stacks of two independent replicates and bars indicate standard deviation. Statistically significant differences between groups were calculated by unpaired *t*-test and marked with one asterisk (*P* < 0.05)

To study the effect of FCS on biofilm architecture, biofilms of a fluorescent derivative of *N. meningitidis* strain HB-1 Δ*nalP* were formed on glass in TSB supplemented or not with 1% FCS and visualized by confocal microscopy. After 1 h, the biofilms formed in the absence of FCS were constituted of compact microcolonies of different sizes and small clusters (Figure 3a). The biofilm biomass covered about ~70% of the surface of the substratum. After 12 h, the biofilm was organized in large clusters of high volume that interacted with each other with small intervening spaces within the biomass (Figure 3a). The biomass covered about 90% of the substratum. These observations suggest that the biofilm in this strain is developed by expansion of initial clusters and cluster interaction. Analysis of 1-h-old biofilms formed in the presence of FCS revealed only few small microcolonies on the substratum, which were separated from each other. Together, they covered less than 5% of the surface of the substratum. After 12 h, biofilms developed with a highly heterogeneous architecture constituted of separate microcolonies and clusters with irregular intervenient spaces (Figure 3a). The biomass covered about 30–60% of the surface area. To gain more insight into the structure of these biofilms, 12-h-old biofilms were analyzed with COMSTAT software and statistically compared. As expected, the biomass, which includes both live and dead bacteria in the biofilm, was significantly reduced in the presence of FCS (Figure 3b). Also, the biofilm thickness was reduced (Figure 3b), but this difference was not statistically significant due to large variation within the biofilm. The roughness coefficient of the biofilms was significantly higher when FCS was added to the cultures, indicating that FCS alters the interbacterial interactions within biofilms. Thus, these analyses demonstrate that FCS prevents the attachment of bacteria to the substratum and alters the biofilm architecture.

### FCS affects the binding of NHBA and α-peptide to DNA

**Figure 4.**
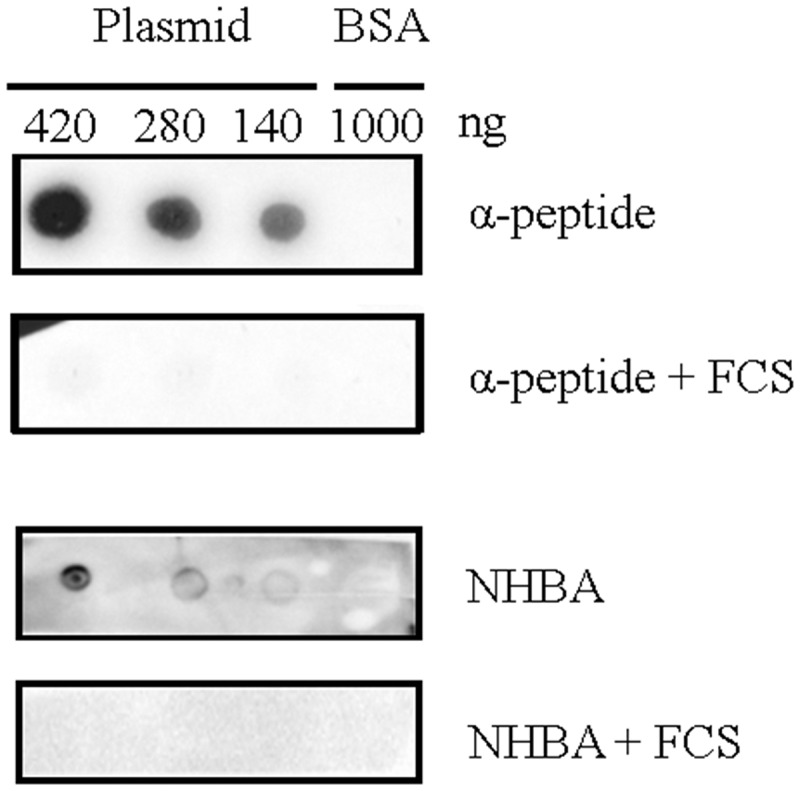
**Far dot-blotting assay probing the binding of recombinant α-peptide of IgAp and NHBA in the presence or absence of FCS to DNA**. Various amounts of the linearized plasmid pETx507_AutAp and, as a control, of 1000 ng of BSA were spotted onto a membrane. The membrane was then blocked and subsequently incubated with recombinant NHBA or α-peptide of IgAp, which were first incubated or not with FCS. The binding of the peptides to the DNA on the membrane was analyzed by immune detection using anti-α-peptide or anti-NHBA antibodies

Binding of eDNA is the basis for the initiation of biofilm formation in HB-1 and its *nalP* -mutant derivative [12]. Thus, we tested whether the binding of NHBA and α-peptide to DNA is affected by FCS. To this end, recombinant α-peptide and NHBA were produced in *E. coli* and purified, and their ability to bind DNA was tested in “far dot-blotting assays”. For these assays, plasmid DNA and, as a control, BSA was spotted onto a nitrocellulose membrane, which was subsequently incubated with the purified proteins. Binding of the proteins to the applied DNA was then detected with specific antibodies. Both proteins were indeed found to bind to the DNA, and not to the membrane-attached BSA (Figure 4). Pre-incubation of the α-peptide or NHBA with FCS inhibited DNA binding (Figure 4). These results indicate that FCS may inhibit biofilm formation by preventing NHBA and the α-peptide to bind DNA.

### Fractionation of serum

The inhibition of the binding of NHBA and the α-peptide to DNA suggested that FCS affects the integrity of these proteins. Serum contains proteases, such as the C3 convertase of the alternative pathway and PKLK, which have previously been shown to cleave meningococcal NHBA [38,39]. However, these proteolyses should not affect the role of NHBA in biofilm formation as cleavage was shown to occur downstream of the arginine-rich region in this protein leaving an N-terminal fragment with DNA-binding capacity at the cell surface. Therefore, we hypothesized that (an)other unknown protease(s) in FCS may cleave NHBA and the α-peptide and thereby inhibit biofilm formation.

To identify the compound(s) that inhibit biofilm formation, FCS was fractionated by SEC followed by AEC (see Figure S2 and expanded results in Supporting information for details). Briefly, the fractions obtained by SEC (Figure S2a) were tested for anti-biofilm activity on strain HB-1 Δ*nalp* (Figure S2b) and for cleavage of IgAp (Figure S2c), and the highest activity fraction was further fractionated by AEC (Figure S2d). The composition of four of the resulting AEC fractions that were found to cleave IgaP differently (Figure S2e) was analyzed by iBAQ for protein identification. iBAQ identified a total of 300 proteins distributed over the four fractions (Table S1 in Supporting information), of which 21 were common to all fractions. In general, protein identification revealed enzymes of metabolic pathways, members of the coagulation pathway, complement factors, released cell-surface membrane proteins, and proteases/peptidases, among many others. Examples of the latter category include dipeptidyl peptidase 1, prolyl endopeptidase FAP, plasminogen, peptidase D, mannan-binding lectin serine peptidase 2, FXII, or PKLK.

### PKLK, plasmin, and FXIIa cleave meningococcal proteins

**Figure 5.**
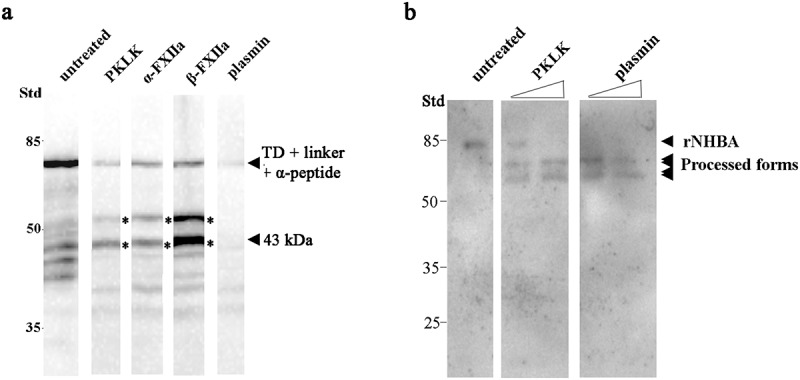
**Cleavage of meningococcal proteins by serum proteases**. (a) Cell envelope preparations of *N. meningitidis* strain HB-1 Δ*nalP* were incubated with 5 µg of PKLK, α-FXIIa, β-FXIIa, or plasmin. Cleavage of the IgAp α-peptide, fused via the linker to the TD, was analyzed by immunoblotting using antiserum against the TD . (b) 0.5 µg of the recombinant NHBA polypeptide was incubated with 8 or 80 ng of PKLK and with 0.032 or 0.32 ng of plasmin and analyzed by immunoblotting using a antiserum against NHBA

Although the iBAQ experiments did not unequivocally identify the protease-mediated cleavage of the α-peptide, we next tested commercially available proteases identified in the serum fractions, i.e. PKLK, FXII, and plasmin, for their ability to cleave the α-peptide of IgAp in cell envelopes of strain HB-1 Δ*nalP*. Of the zymogen FXII, both activated processed forms, α-FXIIa and β-FXIIa, were tested. All these enzymes reduced considerably the amount of intact α-peptide and generated various degradation products (Figure 5a). Plasmin degraded the polypeptide drastically without generating many fragments detectable with our antiserum on the blot (Figure 5a). To assess cleavage of NHBA, we employed the recombinant NHBA polypeptide produced in *E. coli* because our antiserum cross-reacted with several proteins of *N. meningitidis* (Figure S3). This recombinant protein has a predicted molecular mass of 42.7 kDa, but showed a deviant mobility on SDS-PAGE gels (Figure 5b). Note that also the native protein revealed an deviant mobility on gel (Figure S3). This band was not present in the corresponding *nhbA* mutant and heavily processed by NalP in HB-1 (Figure S3). An altered mobility of this protein was also detected by other groups [17]. Of the proteases mentioned above, only PKLK and plasmin cleaved the recombinant NHBA, each producing two bands of about 69 and 59 kDa (Figure 5b), respectively. Both fragments were also detected when an antibody against the N-terminal His-tag was used. Together, these data demonstrate that PKLK, FXII, and plasmin, found in our list of potentially relevant proteases, cleave key proteins involved in meningococcal biofilm formation.

### *PKLK and plasmin inhibit biofilm formation of* N. meningitidis *and* B. pertussis

**Figure 6.**
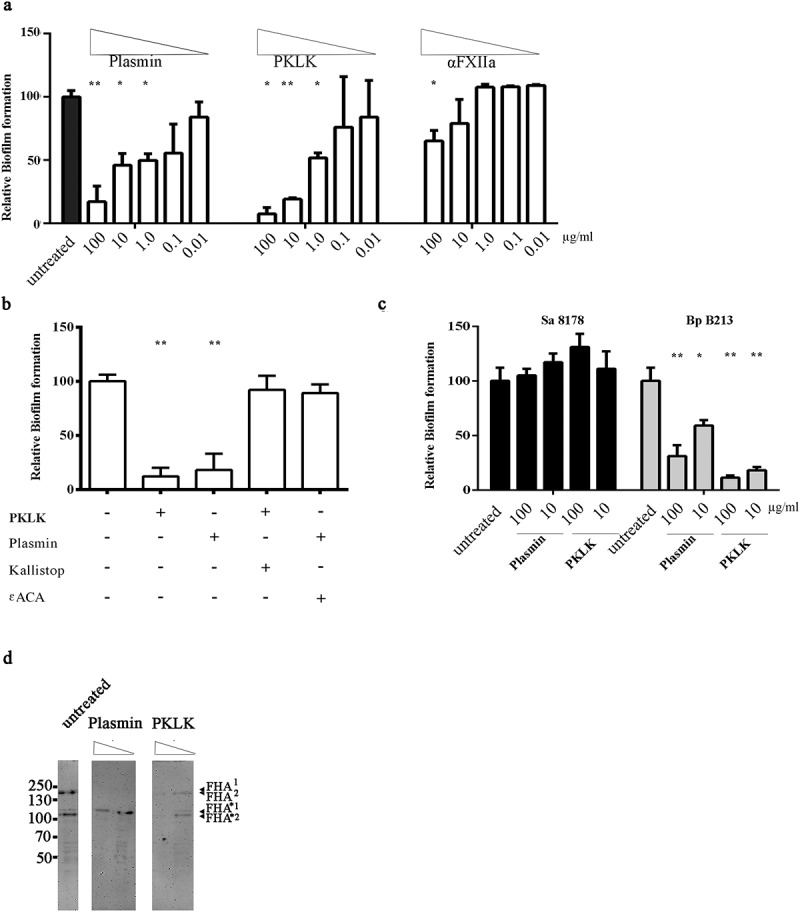
**Inhibition of biofilm formation by serum proteases**. (a) Biofilms of HB-1 Δ*nalP* were formed under static conditions in presence of the purified proteases at the different concentrations indicated. (b) Biofilms were studied of HB-1 Δ*nalP* in the presence of 100 of PKLK or 10 µg/ml of plasmin, respectively, which were either preincubated or not with 50 µM Kallistop or 200 mM ɛACA, respectively. (c) Impact of different concentrations of plasmin and PKLK on biofilm formation of *S. aureus* strain 8178 and *B. pertussis* strain B213. In panels **a–c**, values are given as relative to the no-treatment control, which was set at 100%. Statistically significant differences for each treatment relative to the untreated control are marked with one (*P* < 0.05) or two (*P* < 0.005) asterisks (unpaired *t*-test). (d) Whole cells of *B. pertussis* strain B213 were incubated with 8 or 80 ng of PKLK or with 0.032 or 0.32 ng of plasmin and analyzed by immunoblotting using anti-FHA monoclonal antibodies

We next investigated if the proteases that cleave NHBA and/or the α-peptide of IgAp inhibit biofilm formation. Indeed, PKLK and plasmin drastically decreased biofilm formation in *N. meningitidis* strain HB-1 Δ*nalP* in a dose-dependent manner (Figure 6a). Also, α-FXIIa reduced biofilm formation but only to a lower extent (about 40%) at the highest concentration tested. The biofilm-inhibiting activity of plasmin and PKLK was blocked when the proteases were pre-incubated with their inhibitors ɛACA and Kallistop, respectively (Figure 6b). However, when we added both inhibitors separately or simultaneously to FCS, this did not affect the biofilm-inhibiting activity, which suggests that inhibition of biofilm formation is mediated by multiple factors, including PKLK and plasmin, which is in agreement with the high proteolysis of IgAp by serum fractions (Figure S2c, S2e). We analysed also whether PKLK and plasmin inhibit biofilm formation in two other bacterial species tested in our initial experiments, *B. pertussis* and *S. aureus*. Both proteases indeed inhibited biofilm formation of *B. pertussis* strain B213, but not of *S. aureus* strain NCTC 8178 (Figure 6c).

*B. pertussis* produces a filamentous hemagglutinin called FHA, which is essential for biofilm formation by mediating cell–cell and cell–substratum interactions [40]. Thus, we hypothesized that FHA could be cleaved by PKLK and plasmin. FHA has a molecular weight of 220 kDa and derives from a larger 367-kDa precursor called FhaB through proteolysis mediated by CtpA and SphB1 [41]. Anti-FHA antibodies detected two high-molecular-weight bands of 250 kDa (FHA^1^) and of 240 kDa (FHA^2^) in whole-cell lysates of strain B213 (Figure 6d), corresponding to mature FHA forms (Figure 1), two prominent truncated forms of 115 kDa (FHA^*1^) and 105 kDa (FHA^*2^) (Figure 6d), which could possibly correspond to FHA fragments cleaved within an Arg-rich segment (Figure 1), and several additional degradation products of minor intensity (Figure 6d). Such FHA degradation pattern has been reported previously [41]. To determine if serum proteases target FHA, whole cells of strain B213 were incubated with plasmin or PKLK, and FHA integrity was evaluated by Western blotting. Interestingly, plasmin completely degraded both FHA^1^ and FHA^2^ in a dose-dependent manner, and a degradation pattern was also evident at low concentration. In contrast, the FHA*^1^ form was resistant to plasmin (Figure 6d). PKLK degraded both FHA and FHA* species in a dose-dependent manner and generated several degradation products, but in contrast to plasmin, the degradation of mature and truncated forms was similar (Figure 6d). These observations demonstrate that FHA is indeed targeted by plasmin and PKLK, which may explain the inhibition of pertussis biofilm formation by both enzymes and FCS.

## Discussion

Biofilm formation is a strategy commonly used by pathogenic bacteria to resist the host immune system. Understanding the host response to biofilms may open novel avenues to improve outcomes of infectious diseases. Here, we found that FCS inhibits initiation of biofilm formation of several human pathogens that cause systemic or local infections. Several serum proteases were identified that inhibit biofilm formation in *N. meningitidis* and that cleave NHBA and/or the α-peptide of IgAp, two cell surface-exposed proteins that have critical roles in the initiation of biofilm formation in this model organism [12]. Interestingly, at least some of these proteases inhibited biofilm formation also of *B. pertussis*, indicating a broader activity against various pathogenic bacteria.

PKLK and FXIIa are components of the contact system, while plasminogen participates in the fibrinolysis system [42]. Kallikreins comprise a family of secreted serine proteases. There are two types of kallikreins: PKLK, produced in pancreas and present in blood, and tissue kallikreins (KLK), which were further classified in 15 isoforms (designated 1–15, e.g. KLK1) [43]. Our Western blotting assays revealed that PKLK processes NHBA resulting in two visible fragments that migrated at 69 and 59 kDa. KLK1 and PKLK have preference for Arg-Ser sequences [44]. Recently, Pantano et al. reported that both PKLK and KLK1 cleave NHBA of *N. meningitidis* strain MC58 [39]. N-terminal sequencing of the released fragment demonstrated that it starts with a Ser_306_ located in the Arg-rich segment [R_298_RSARSRR**S_306_**] (Figure 1). Thus, the 69-kDa form found in our Western blotting assays could correspond with the cleavage of Arg-Ser sequences located at the Arg-rich region. As indicated above, this region contains three Arg-Ser sequences. It is entirely conceivable that PKLK cleaves at all these sites, resulting in C-terminal truncation of the N-terminal fragment and the loss of several Arg residues at the C-terminus of this fragment leading to the loss of DNA-binding activity and biofilm formation. Additionally, PKLK cleaves upstream of the arginine-rich region. Although PKLK has preference for Ser at the P1´ position, it can also cleave other Arg-X sequences. One Arg-Val sequence [F_202_GRVD_206_] could be a hypothetical target for PKLK. Actually, PKLK can activate plasminogen by cleaving an Arg-Val bond. Cleavage of rNHBA at this site could explain the 59-kDa fragment found in our assays. Obviously, the released fragment would contain the Arg-rich segment that will be further processed at Arg-Ser sequences by PKLK or by other proteases. The longer incubation times used by Pantano et al. [39] (overnight) as compared to ours (30 min) cannot explain why these authors did not detect two fragments in their assays. Notably, here and in Pantano’s work, different recombinant polypeptides were used, the folding of which could be also different. Protein folding can restrict the access of proteases to their target sites. Thus, folding could cause a different accessibility of the protease to the Arg-Val sequence in the recombinant polypeptides.

Apart from NHBA, also the α-peptide of IgAp can contribute to biofilm formation, but this depends on the *nalP* expression status [12]. When NalP is not produced, the α-peptide, which contains one or several positively charged segments that could bind eDNA, is exposed at the bacterial cell surface bound via a linker to the TD (Figure 1), which is an integral outer-membrane β-barrel protein. We observed that PKLK also cleaves the surface-exposed α-peptide from its membrane anchor (Figure 5). The α-peptide of strain HB-1 contains one Arg-rich segment [P_1172_KRRNRR_1178_], which functions as a nuclear localization signal (NLS), at its C terminus (Figure 1). Cleavage at this Arg-rich segment would release the α-peptide, while a fragment consisting of the β-barrel plus the linker domain, consistent with the 43-kDa fragment detected in the Western blotting assays, would be retained in the cells. The retained fragment lacks the presumed DNA-binding motif.

Plasmin is a serine protease generated from plasminogen. We found that plasmin also cleaves NHBA. The generated fragments showed a similar size as those generated by PKLK, suggesting that this protease also removes the DNA-binding domain. Additionally, plasmin was more active than PKLK in degrading NHBA, as much lower concentrations of plasmin were needed to get similar degradation. Also, plasmin degraded the IgA polypeptide consisting of α-peptide, linker, and TD further than PKLK did, indicating that plasmin could recognize many more cleavage sites than PKLK. So far, the cleavage of meningococcal proteins by plasmin has not been reported yet. Both forms of FXII, α-FXIIa and β-FXIIa, also cleaved IgAp but not NHBA. FXIIa has preference for Arg-Val sequences, but can also cleave at other Arg-X sequences. For example, it cleaves the natural substrates Factor XI and prekallikrein both at an Arg-Ile sequence [45]. During self-activation, it cleaves itself at three sites, Arg-Asn, Arg-Leu, and Arg-Val. Most likely, it will also cleave in the Arg-rich NLS at the C terminus of the α-peptide, generating the same prominent 43-kDa fragment as observed after PKLK treatment. In summary, the activity of the enzymes is in agreement with the loss of DNA binding capacity of IgAp and NHBA when incubated with FCS (Figure 4).

In previous work, we have demonstrated the importance of eDNA, NHBA and the α-peptide in the biofilm formation process of *N. meningitidis* [12]. Also *N. gonorrhoeae* uses eDNA for biofilm formation [46], and NHBA was recently shown to play also a critical role in microcolony formation [47]. FCS inhibited the formation of biofilms by strain FA1090 (Figure 2). As in the meningococcus, FA1090 NHBA contains an Arg-rich segment [_240_RSARSRRSL_248_] and one Arg-Val sequence [_218_ADRVKK_223_] located upstream of the Arg-rich segment (see above), which could be targets for PKLK. As *N. gonorrhoeae*, in contrast to *N. meningitidis*, does not produce NalP, the Arg-rich region of all NHBA molecules remains surface exposed. Besides, IgAp of *N. gonorrhoeae* FA1090 contains four NLS in the α-peptide, which could be involved in biofilm formation by binding eDNA. Cleavage at the most C-terminal Arg-rich NLS of the IgAp [_1210_PKRRGRRS_1217_] will remove all four stretches from the cell surface. This segment could be targeted by PKLK, plasmin, and/or FXII, which together could explain the sensitivity of biofilm formation by *N. gonorrhoeae* for FCS (Figure 1). In *Neisseria*, NHBA is probably very relevant on the adherence to epithelial tissues of the human mucosa [19,47]. Adherence is the first step in bacterial colonization and, therefore, these adhesins are considered virulence factors. However, microcolonies , which resemble biofilms, can also be formed on the endothelial surfaces of the vascular system. It has been considered that type 4 pili are the only important bacterial factor in this process [5], which makes sense if, indeed, NHBA and the α-peptide are cleaved from the cell surface by serum proteases. It will be interesting to test whether type 4 pili and many other virulence factors used for the bacteria to cause septicaemia are resistant to the serum proteases.

Besides inhibiting biofilm formation, the cleavage of NHBA and the α-peptide of IgAp by serum proteases may have other consequences for the biology of the infection. The NLS present in the α-peptide has been shown to target the nucleus of transfected cell lines [48]. When the released α-peptide remains fused to the IgAp protease domain after NalP-mediated processing, the protease is targeted to the nucleus where it cleaves the NF-κB component p65/RelA, thus modulating the innate immune response [49]. Proteolytic cleavage of the released protein at the NLS by serum proteases would prevent this immune evasion mechanism. Additionally, NalP cleaves NHBA releasing the C-terminal fragment including the Arg-rich segment into the environment, where it alters the permeability of endothelial cell layers and thereby contributes to vascular leakage [20]. PKLK or plasmin could have a protective role for the host by removing the Arg-rich motif from the released fragment. In the absence of NalP, the α-peptide and intact NHBA remain at the bacterial cell surface where they can bind heparin [17,31], presumably through the positively charged Arg-rich stretches. Heparin interacts with proteins of the complement system including factor H, C4b-binding protein, and C1 inhibitor [50]. The binding of these factors via heparin to the bacteria has been proposed as a mechanism to inhibit complement-mediated killing of unencapsulated bacteria. Also, heparin bound to the bacterial surface might bind vitronectin. Mammalian cells express vitronectin receptors, which can recognize vitronectin with attached cells allowing bacterial uptake [51].

In this work, we used *N. meningitidis* as model microorganism, but we observed that FCS also affects the capacity of several other pathogens to form biofilms (Figure 2d). In *B. pertussis*, also pure PKLK and plasmin inhibited biofilm formation (Figure 6c). The *Bordetella* genus comprises pathogenic microorganisms, including *B. pertussis* and *B. bronchiseptica*, which cause infections in the lower respiratory tract of humans and animals, respectively. Both species produces a filamentous hemagglutinin called FHA, which is secreted via the type V secretion system and remains associated with the bacterial cell surface. Pertussis FHA is essential for biofilm formation by mediating cell–cell and cell–substratum interactions [40]. Like NHBA and the α-peptide of IgAp of *Neisseria*, FHA binds heparin [52], possibly via a conserved Arg-rich segment (Figure 1), and it is, therefore, likely to bind also eDNA. Indeed, eDNA is relevant for biofilm formation of *B. pertussis* [53]. Interestingly, PKLK and plasmin cleave FHA, premumably at the Arg-rich segment, which contains putative cleave sites for both enzymes. Consequently, bacteria can lose their eDNA-binding capacity upon cleavage. However, some *B. pertussis* strains, such as strain B213, may use an eDNA-independent biofilm-forming strategy (Figure 2c), where the contribution of FHA to direct interbacterial interactions may be more relevant than the eDNA-binding capacity. *In-vivo* studies have shown that FHA is required for the infection and persistence of *Bordetella* in the lower respiratory tract of animals and humans by playing roles in adhesion and immunomodulation [54,55]. Cleavage of FHA by host proteases could help to prevent bacterial colonization and the establishment of the infection. Interestingly, the C1 inhibitor binds *B. pertussis* through Vag8 [56] attenuating its inhibitory function on FXIIa. FXIIa activates PKLK with subsequent activation of (i) the kallikrein-kinin pathway, which ends up with the activation of an inflammatory response, and (ii) the fibrin degradation pathway, including plasmin activation. Thus, besides being participants of activation routes addressed to pathogen elimination, PKLK and plasmin are direct effectors of *Bordetella* clearance. In a broader perspective, considering that FCS inhibits biofilm formation of different pathogens (Figure 2), it would be interesting to know whether the same proteases identified here also inhibit biofilm formation in the other pathogenic bacteria and what the targets for the proteases are in these bacteria. But, in contrast to *N. meningitidis* and *B. pertussis*, PKLK and plasmin did not have activity on *S. aureus* biofilms (Figure 6b). Probably, some pathogens have evolved strategies to escape the host protease activities. So far, our work opens new avenues for understanding the broad spectrum of host immune defense mechanisms against pathogens. Besides, the application of enzymes to fight biofilms has been broadly proposed [3,57–58]. Although the practical traits of the proteases found here to prevent biofilm formation requires further thorough investigations, our initial results suggest that they could be an tool for therapeutic antibiofilm treatment.

## Supplementary Material

Supplemental Material

## Data Availability

The authors confirm that the data supporting the findings of this study are available within the article and its supplementary materials.
