# Supplementary material for: Serum proteases prevent bacterial biofilm formation: role of kallikrein and plasmin
Source: Virulence. 2021 Dec 14;12(1):2902–17. doi: 10.1080/21505594.2021.2003115 (PMC8677018; doi:10.1080/21505594.2021.2003115)
Supplement: Supplemental Material [file KVIR_A_2003115_SM7025.pdf]

Supporting information

Serum proteases prevent bacterial biofilm formation: role of kallikrein and plasmin

Jesús Arenas, Zalan Szabo, Jelle van der Wal, Coen Maas, Tahira Riaz, Tone Tønjum, Jan Tommassen

Figure S1. Impact of serum on biofilm formation.

Expanded results of serum fractionation

Figure S2. Fractionation of FCS.

Figure S3. Production of NHBA in *N. meningitidis* strain HB-1 and its *nalP*-mutant derivative.

Table S1. Proteins identified by iBAQ in four fractions resulting from fractionation of fraction 10 from EAC.

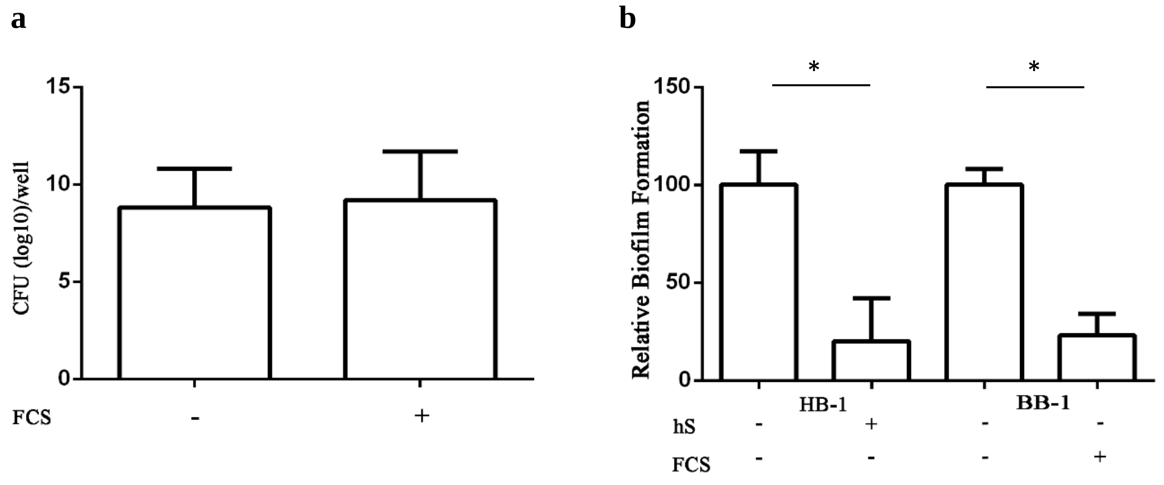

Figure S1. Impact of serum on biofilm formation. (**a**) Effect of foetal calf serum (FCS) on viability of *N. meningitidis* strain HB-1. Bacterial cultures in TSB medium were incubated for 1 h in 24-wells plates in the presence or absence of 5% FCS as described for biofilm formation. Then, the colony-forming units (CFU) in the culture medium were determined by plating on GC medium and overnight incubation. (**b**) Impact of human serum (hS) or FCS on biofilm formation of *N. meningitidis* strains HB-1 and BB-1. Biofilms were formed in 24-wells plates and quantified after growth in TSB with or without 5% serum for 1 h or 8 h for HB-1 and BB-1, respectively. Values are given as relative to no treatment (for each strain), which was set at 100. Statistically significant differences ($P < 0.05$) are marked with one asterisk.

Expanded Results of Serum Fractionation

To identify the compound(s) that inhibit biofilm formation, FCS was fractionated by size-exclusion chromatography (SEC). Recovered fractions from SEC separation were analysed by SDS-polyacrylamide gel electrophoresis (SDS-PAGE), which showed the presence of proteins in fractions 6 to 16 with good separation of the proteins according to size (Figure S2a). The fractions were tested on strain HB-1 Δ*nalP* for inhibition of biofilm formation, and the highest activity was found in fractions 9, 10, 12, and 13 (Figure S2b). To investigate whether these fractions contained protease activity against the α-peptide of IgAp, they were incubated with whole cells of strain HB-1 Δ*nalP* in which the α-peptide is present at the cell surface attached to the linker and TD of IgAp as, together, a 75-kDa polypeptide [31]. FCS cleaved the α-peptide from this protein yielding a 43-kDa fragment corresponding to the linker and the TD [31], which was detected with antiserum directed against the TD (Figure S2c). SEC fractions 9 to 12, which showed anti-biofilm formation activity (Figure S2b), also produced this 43-kDa fragment in contrast to the other SEC fractions (see representative results in Figure S2c). We did not test the integrity of NHBA in these assays. Although we did detect NHBA in whole cell lysates of HB-1 and its *nalP*-mutant derivative with the anti-NHBA antiserum, also many cross-reacting proteins were observed (Figure S3).

SEC fraction 10 revealed the highest biofilm-inhibitory activity and cleavage of the α-peptide and was therefore further fractionated by AEC. The protein content of the 27 fractions recovered from the column was evaluated by SDS-PAGE. Fractions 12 to 17 had the highest protein content (Figure S2d). Incubation of HB-1 Δ*nalP* with fractions 8 to 26 showed various cleavage products of the cell-surface-associated α-peptide of IgAp, dependent on the fraction used (see representative results in Figure S2e). This data indicates that the activity of FCS on the α-peptide of IgAp is caused by several proteases. To identify them, we analyzed four fractions, i.e. fractions 8, 13, 16, 24, which yielded different cleavage patterns in the assay above (Figure S2e), by intensity-based absolute quantification (iBAQ) for protein identification. The MaxQuant software engine uses a list of known potential contaminants that are often identified during mass spectrometry analyses. These potential contaminants were then removed during filtering in Perseus (see material and methods section). Finally, we identified a total of 300 proteins distributed in the four fractions (Table S1), of which 21 were common to all fractions.

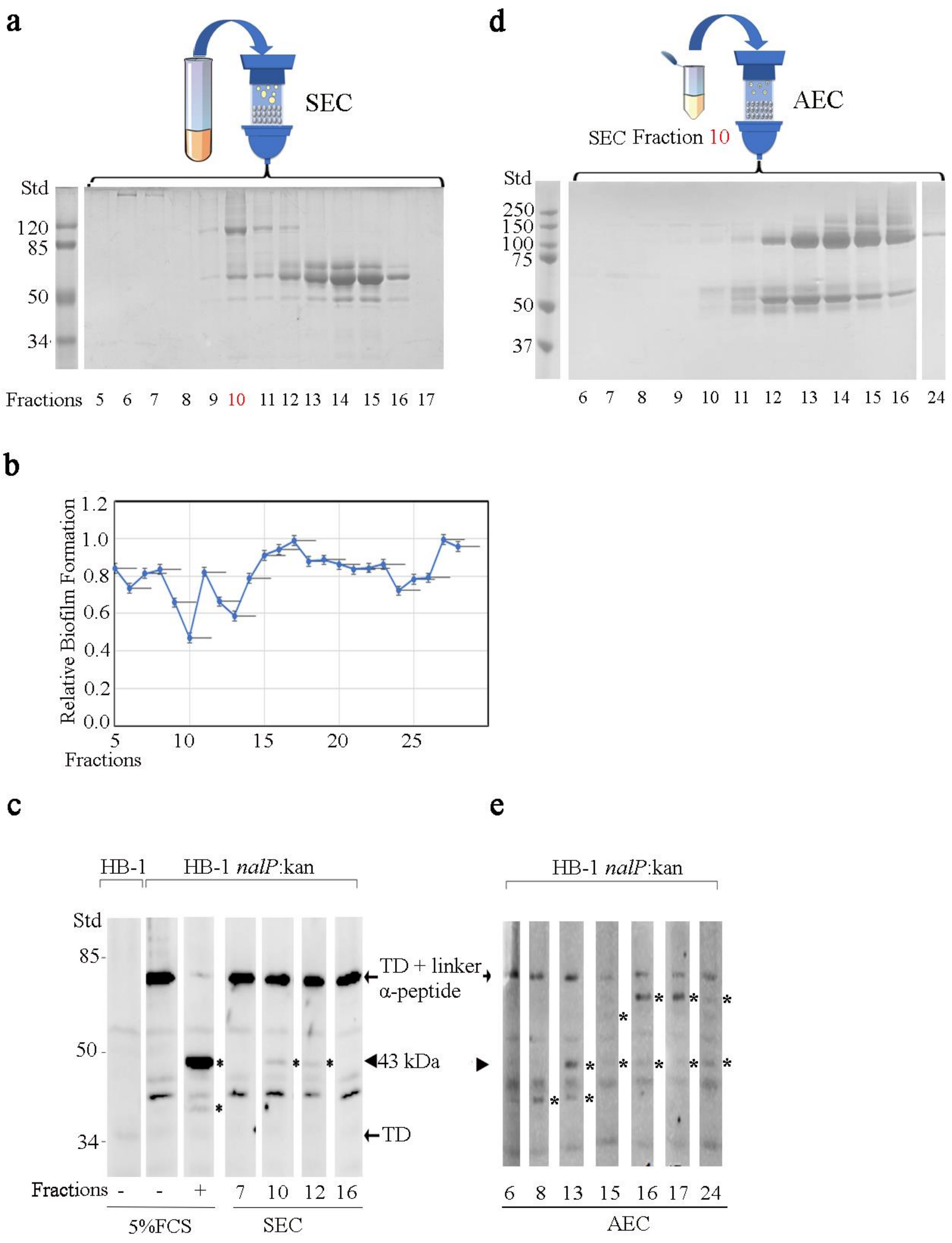

Figure S2. Fractionation of FCS. The components of FCS were separated by SEC and the resulting fraction 10 was further fractionated by AEC. (**a, d**) SDS-PAGE analysis of fractions 5 to 17 obtained from SEC (panel **a**) and of fractions 6 to 16 and 24 obtained from AEC (panel **d**). (**b**) Inhibition of biofilm formation of strain HB-1 Δ*nalP* by SEC fractions. Cultures of HB-1 Δ*nalP* were mixed with SEC fractions, and biofilm formation was quantified after 2 h by crystal-violet staining. Values are given as relative to no treatment, which was set at 1.0. Average and standard deviation (error bars) of two independent experiments are given. (**c, e**) Cleavage of the cell-surface-exposed α-

peptide of IgAp by FCS components. Whole cells of *N. meningitidis* strain HB-1 and its *nalP*-mutant derivative were incubated with 5% FCS or with FCS fractions obtained from SEC (panel **c**) and AEC (panel **e**) as indicated. After incubation, bacterial cells were centrifuged, and the pellet was analysed by SDS-PAGE and Western blotting with antiserum directed against the TD of IgAp. The positions of the relevant polypeptides are indicated at the side of the blots and cleavage products are marked with asterisks.

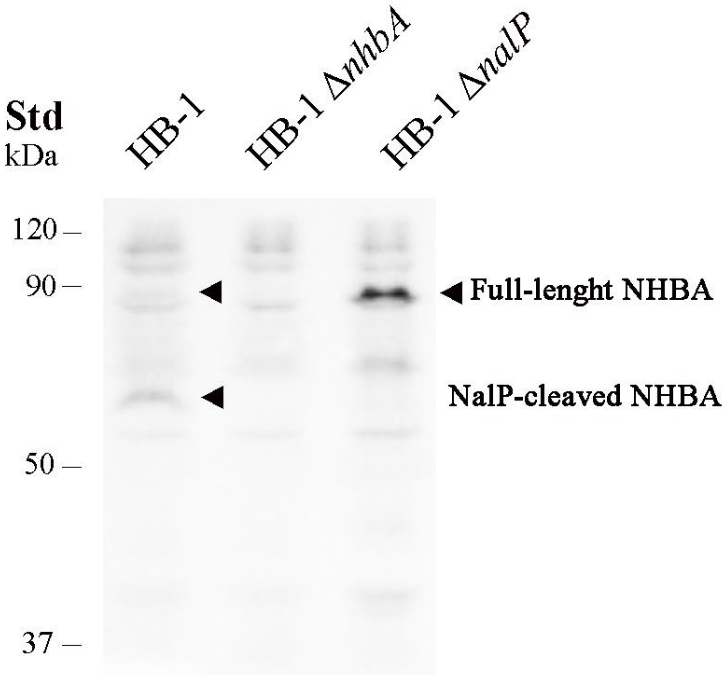

Figure S3. Production of NHBA in *N. meningitidis* strain HB-1 and its *nalP*-mutant derivative. Immunoblots of whole-cell lysates of *N. meningitidis* strain HB-1 and its *nalP*- and *nhbA*-mutant derivatives were probed with anti-NHBA polyclonal antiserum. The positions of NHBA and the N-terminal fragment generated by NalP-mediated cleavage are indicated at the right. The positions of molecular weight standards (Std) are indicated at the left. Although the calculated molecular mass of NHBA is 50.5 kDa, NHBA migrates for unknown reasons with an apparent molecular weight of 86.2 kDa in our gels.

Table S1. Proteins identified by iBAQ in four fractions resulting from fractionation of fraction 10 from EAC. Only fractions 8, 13, 16 and 24 were tested. Regular FCS contaminants were extracted (data not shown).

**EAC fraction (iBAQ Log2)**				**Protein ID**	**Protein name**
**13**	**16**	**24**	**8**		
26,04856	25,54937	20,41256	22,68676	O46375	Transthyretin
17,77398	18,58075	16,59313	21,34841	G5E604	Uncharacterized protein
21,66566			21,097	Q5E9F8	Histone H3.3
23,35421			20,94328	P84227	Histone H3.2
			19,72802	P68432	Histone H3.1
18,87346	18,27258	18,64054	18,96282	F1MLW8	Uncharacterized protein
21,39032	22,5232	17,43705	17,257	Q2KJ63	Plasma kallikrein
			17,06844	A2VDR3	Cerebellin 4
			17,03176	A7MBG9	Eukaryotic translation initiation factor 4H
18,05086	19,62064	13,91017	16,96319	P68103	Elongation factor 1-alpha 1
			16,31897	P31081	60 kDa heat shock protein, mitochondrial
21,675	21,92713	15,79353	14,25185	Q5NTB3	Coagulation factor XI
17,68586	17,57932	11,84082	13,68113	Q3ZCJ8	Dipeptidyl peptidase 1
17,52734	19,25133	13,53843	12,64876	Q28106	Contactin-1
	22,14143	14,73962	12,38683	P63258	Actin, cytoplasmic 2
17,9607	20,39822	15,72739	11,92715	A5D984	Pyruvate kinase
19,61301	21,71406	16,09133	11,31328	A5D7B7	Prolyl endopeptidase FAP
14,59485	15,48947	10,24484	10,82265	Q29451	Lysosomal alpha-mannosidase
		15,2658	10,61967	P81947	Tubulin alpha-1B chain
18,02545	17,55637	12,04354	10,13738	F1MWK8	Protein tyrosine kinase 7 (inactive)
12,14281	14,43306	9,816008	7,823112	F1MSR8	Collagen alpha-1(II) chain
25,35696	25,04852			F1N6Y1	Glucosidase II alpha subunit
24,96983	26,74842	20,01713		F1MYX2	Apolipoprotein M
24,22288	22,97222	14,46448		Q3SZV7	Hemopexin
24,09023	23,45814	12,98911		Q7YS82	Myoblast determination protein 1
22,93121	21,88569	16,65606		E1BI98	Collagen type VI alpha 1 chain
22,576	22,25931	17,57112		Q6URK6	Cadherin-5
22,55056	22,41903	15,46132		Q0VC51	Interleukin 1 receptor accessory protein
22,19304	23,52981	18,09826		Q32KY0	Apolipoprotein D
21,89792	23,27909	15,79723		Q32L76	Serum amyloid A-4 protein
21,84019	21,21798	13,93719		Q5E9F7	Cofilin-1
21,6865	19,86932	14,76404		F1MX87	Complement C8 alpha chain
21,50857	20,24632	14,27961		A5PJT7	ECM1 protein
21,18035	20,08784	9,681256		G3N019	Transmembrane protein 132C
21,08531	19,82836	12,60117		Q24K22	Hepatocyte growth factor-like protein

21,02168	20,26762	16,11902		Q2YDI9	Ferritin, mitochondrial
20,98169	19,70786	12,38567		Q9GKR3	Vascular cell adhesion molecule-1 7D variant
20,94935	21,00154			Q0VCH9	GLI pathogenesis-related 2
20,72991	24,27412	16,75042		P19035	Apolipoprotein C-III
20,51788	20,17937	15,62819		Q5I597	Betaine--homocysteine S-methyltransferase 1
20,44233				A8E4L9	SYNC protein
20,35262	19,90138	16,01083		F6Q234	Peptidase D
20,25569	23,46625	18,92958		G3N0B6	Vasorin precursor
20,22647	19,58709	14,0563		F1MKP6	Cadherin-13
20,11312	19,51466	14,1148		Q2NKV6	Metallothionein
19,8483	18,59478	11,65906		F1MGX7	Immunoglobulin superfamily DCC subclass member 4
19,6512	19,20915	15,06886		F1MW44	Coagulation factor XIII A chain
19,63894	22,20771	13,62377		P19534	Cadherin-2 (Fragment)
19,59562	22,2674	16,56674		A2VE41	EGF-containing fibulin-like extracellular matrix protein 1
19,56765	17,87057			E1BEV1	Coiled-coil domain containing 17
19,53812	20,58793	13,46097		E1BLA8	Golgi membrane protein 1
19,53249				A8QQF2	MCAM protein
19,51507	19,1097	9,76303		F1MS00	Proto-oncogene tyrosine-protein kinase receptor Ret
19,4966	19,24917			Q29RY7	Fibroleukin
19,45439	21,6856	12,05464		P55906	Transforming growth factor-beta-induced protein ig-h3
19,42095	21,76822	19,65288		P00745	Vitamin K-dependent protein C (Fragment)
19,39638	21,52229	14,94444		F1N0X0	Protocadherin 12
19,39178	19,50392			P13384	Insulin-like growth factor-binding protein 2
19,35182	16,98837			Q28125	Intercellular adhesion molecule 3
19,32085	19,71287	15,06979		A6QLL8	Fructose-bisphosphate aldolase
19,26128	21,03557	14,46754		Q7YRD0	RNA-binding protein PNO1
19,25997	18,14709	14,56159		P04815	Spleen trypsin inhibitor I
19,16047	20,72974	12,84333		G8JKZ8	Thioredoxin
19,15869	15,35996			Q3MHQ1	Amine oxidase
19,15462	22,1861	16,12876		Q58DL9	Phospholipid transfer protein
19,09648	18,11304	11,60038		A3KLR9	Superoxide dismutase [Cu-Zn]
19,06626	21,2972	17,74793		E1B805	Uncharacterized protein
18,99386	22,32767	15,18793		F1N7F9	Calcium voltage-gated channel auxiliary subunit alpha2delta 1
18,96327	18,39125			P07456	Insulin-like growth factor II
18,9558	18,7346	10,85922		A8YXZ2	C8G protein
18,86233	16,92833			F1MNV5	Kininogen-1
18,84953	19,12609			P52175	Nucleoside diphosphate kinase A 2

18,75094	20,57653			Q9BGI3	Peroxiredoxin-2
18,71008	17,21314	11,23548		F1MUK3	Insulin-like growth factor-binding protein 6
18,69374	18,82814	16,30241		F1MY85	Complement C5a anaphylatoxin
18,68031	17,18592	8,069208		P08169	Cation-independent mannose-6-phosphate receptor
18,67028	18,7787	14,68458		F1N726	Glycoprotein 2
18,65561	18,71455	11,75514		E1BB91	Collagen type VI alpha 3 chain
18,63243	19,25778	15,0746		Q2KJ64	Arginase-1
18,57917	23,46114	17,74162		F1MJZ4	Uncharacterized protein
18,56692	19,41361	12,62776		P09487	Alkaline phosphatase, tissue-nonspecific isozyme
18,52784	17,87879			A7MBB0	VCAM1 protein
18,44431	20,49443	15,75546		Q29423	CD44 antigen
18,41361	19,83516	12,99069		G3X755	Plexin B2
18,37071	19,02185	14,92889		F1MHE4	Plasmalemma vesicle associated protein
18,36568	20,01386	15,30135		P15781	Pulmonary surfactant-associated protein B
18,36286	19,2969	14,94522		A7Z067	CSF1R protein
18,33921	17,46292			P98140	Coagulation factor XII
18,29208	18,24679	10,74987		E1BCU8	Uncharacterized protein
18,22473				P04985	Elastin
18,12979	15,85248			E1BKL4	Cartilage intermediate layer protein 2
18,10395				Q865B7	Peroxisome proliferator-activated receptor gamma coactivator 1-alpha
17,96104	20,65988	15,97327		Q3T0S5	Fructose-bisphosphate aldolase B
17,89285	17,33534			Q95132	Intercellular adhesion molecule 1
17,89149	19,64013	14,85681		F1MM32	Sulfhydryl oxidase
17,85811	15,97795	11,94314		P20959	Insulin-like growth factor-binding protein 3
17,85739	22,15193	16,2534		Q2KIW4	Lecithin-cholesterol acyltransferase
17,81228	17,15205			Q9BH13	CD166 antigen
17,71512	20,99085	17,87538		E1BFN6	Dihydropyrimidinase
17,65641				E1BKU3	Uncharacterized protein
17,61296	19,5184	13,84725		Q2KJH6	Serpin H1
17,49997	17,0897			A4FUY2	ATF6B protein
17,48432	18,31871			F1MU18	Oncostatin M receptor
17,47896	19,94949			P80109	Phosphatidylinositol-glycan-specific phospholipase D
17,42341	19,74241			Q2TBL2	Cell adhesion molecule 1
17,42103	16,87867	14,33658		Q0P569	Nucleobindin-1
17,38127	22,96208	16,30547		Q58CS8	N-acetylglucosamine-1-phosphotransferase subunit gamma

17,3605	18,96829	13,81898		Q58D31	Sorbitol dehydrogenase
17,35776	20,84644	17,15857		F1N102	Complement C8 beta chain
17,33525	16,56799			E1BKZ1	Glutathione-disulfide reductase
17,23351	18,20514	9,871043		F1MNM2	Phosphatidylinositol-glycan-specific phospholipase D
17,23201	18,93203			F1MKG2	Collagen type VI alpha 2 chain
17,1136	18,35471			E1B7G5	RNA binding motif protein 12B
17,08317	16,77134	10,80872		E1B726	Plasminogen
17,05833	19,9346	14,44644		P00432	Catalase
17,01987	17,30555	13,39741		P33433	Histidine-rich glycoprotein (Fragments)
16,95311	18,47813			E1BPU9	Protein tyrosine phosphatase, receptor type M
16,94661	19,76539	14,14553		Q08E20	S-formylglutathione hydrolase
16,8906	16,67659			P49907	Selenoprotein P
16,72081	13,10893			Q4U5R3	Proteasome activator complex subunit 1
16,70954	16,97029			Q05716	Insulin-like growth factor-binding protein 4
16,5958	15,23493			E1BF27	Solute carrier family 38 member 10
16,55612	14,95497			F1MZL1	Muellerian-inhibiting factor
16,53529				P11116	Galectin-1
16,53515	15,70353			Q5E947	Peroxiredoxin-1
16,52586	21,87222	17,51876		E1BJ49	Mannan binding lectin serine peptidase 2
16,49403	19,08231	14,59613		P10096	Glyceraldehyde-3-phosphate dehydrogenase
16,48445	17,41749			Q28035	Glutathione S-transferase A1
16,4731	20,90139			Q28022	Microfibrillar-associated protein 5
16,30539	23,0845	15,71341		Q3ZC07	Actin, alpha cardiac muscle 1
16,30182	14,33253			F1MUP1	Folate hydrolase 1
16,30173	17,67273	12,6847		Q5E9Z2	Hyaluronan-binding protein 2
16,29442				Q08DQ6	Zyxin
16,20623	16,49134			Q3SZK8	Na(+)/H(+) exchange regulatory cofactor NHE-RF1
16,20172	17,54661			Q3ZBY4	Fructose-bisphosphate aldolase
16,19994	18,70863	13,11263		P00435	Glutathione peroxidase 1
16,18629	15,82667			Q9TTK6	Membrane primary amine oxidase
16,1281	14,82948			Q2HJE5	Protein FAM234A
16,1236	17,1448	11,12011		Z4YHD9	Hepatocyte growth factor receptor
16,1007	15,88028			Q9BGI2	Peroxiredoxin-4
16,09073	18,56454	12,82243		F1MI18	Uncharacterized protein
16,06693				D1Z308	Periostin
16,06217	19,32014	14,42613		Q3ZCI4	6-phosphogluconate dehydrogenase, decarboxylating
16,00064	15,39691			F1MJV6	Adhesion G protein-coupled

					receptor G6
15,89959				A7MB07	ADAM metallopeptidase with thrombospondin type 1 motif 1
15,78139	17,33246	13,10412		F6RQK3	Glutathione S-transferase zeta 1
15,73355	19,26769	16,49812		P22226	Cathelicidin-1
15,7012	19,06918	13,7932		Q0VCP3	Olfactomedin-like protein 3
15,64647	18,2433			A6QQ20	TGOLN2 protein (Fragment)
15,57607	20,78261	16,51063		Q5E9B1	L-lactate dehydrogenase B chain
15,5069	16,98371	12,56138		F1N619	Cadherin-1
15,48753	15,14466			F1MRZ3	Adhesion G protein-coupled receptor G2
15,47044	16,87652			E1BD36	Interleukin 6 signal transducer
15,46522	19,87995	14,95747		P19858	L-lactate dehydrogenase A chain
15,41373	15,1315			F1MFJ3	Protein tyrosine phosphatase, receptor type G
15,38798	14,94901			P02465	Collagen alpha-2(I) chain
15,30574	16,81828	12,02517		Q06805	Tyrosine-protein kinase receptor Tie-1
15,30407	17,47207			Q17QC8	Complement factor properdin
15,27044	19,37382			Q2HJ65	Core histone macro-H2A
15,17867	19,15469	14,54442		F1N3V0	Malic enzyme
15,17196	19,53721	13,89898		E1BJN3	Amine oxidase
15,15806	20,01018	13,47358		F1N759	Platelet-derived growth factor receptor beta
15,13811	18,2307	13,98566		F1N045	Complement component C7
15,06512	16,97118			Q58D84	Follistatin-related protein 1
15,02315	23,20471	20,3309		P02453	Collagen alpha-1(I) chain
14,99037				E1BIR2	Dipeptidase
14,93424	19,44277	13,26617		P61223	Ras-related protein Rap-1b
14,93286	17,0927	11,51747		F1MVP0	ADAM metallopeptidase with thrombospondin type 1 motif 13
14,90426	15,40517			Q2HJB6	Procollagen C-endopeptidase enhancer
14,90181	18,03725	12,60305		Q2HJ49	Moesin
14,88307	18,95748			Q58DC0	Serine/threonine-protein phosphatase CPPED1
14,85199				F1N0I3	Coagulation factor V
14,84662	17,62257			Q0VCA8	3-hydroxyanthranilate 3,4-dioxygenase
14,79791	17,87195	12,57424		F1MYZ4	Semaphorin 4B
14,79025	15,96675	16,75134		E1BGJ5	CD93 molecule
14,78105	14,99396			P54149	Mitochondrial peptide methionine sulfoxide reductase
14,75708	13,4345	9,866398		F1N4C5	Plexin D1
14,55447	13,39888			E1BI82	Uncharacterized protein
14,5235	16,91474	10,33181		B8Y9T0	Cumulus cell-specific fibronectin 1

					transcript variant
14,5073	16,75186	21,27242		Q3SWW8	Thrombospondin-4
14,37565	17,05833	11,67895		Q17QI3	Acetyl-CoA acetyltransferase 2
14,32994	19,76987	11,78966		F1N2K1	Prenylcysteine oxidase 1
14,30521	16,25851			F1N2I5	Carboxymethylenebutenolidase homolog
14,29333	18,03542	15,63132		E1BI02	Fibromodulin
14,17758	18,3047	11,21953		A4IFA6	Immunoglobulin superfamily containing leucine-rich repeat protein
14,09416	14,49035			F1MEW5	Fibulin 7
14,04277	15,94695	11,44874		F1MXR3	ADAMTS like 4
13,89718	18,88745	12,26104		Q3ZC30	Sulfotransferase
13,86254	16,78813	12,97748		Q27975	Heat shock 70 kDa protein 1A
13,79452	15,17071	16,45119		A5D7L1	C-type lectin domain containing 11A
13,77571	22,63803	11,06063		E1BDT3	Chymotrypsinogen B precursor
13,74976	15,77314	16,63863		O18979	Neuroendocrine secretory protein 55
13,74031	14,72983			F1MYZ7	Fumarylacetoacetase
13,72537	14,62577			F1MNS5	Ectonucleotide pyrophosphatase/phosphodiesterase 1
13,65765	16,9016			P42916	Collectin-43
13,64701	17,30715			Q3SZB7	Fructose-1,6-bisphosphatase 1
13,64262	21,28269	9,831323		F1MHP5	Fms related tyrosine kinase 4
13,60756	19,28888	12,85423		P14568	Argininosuccinate synthase
13,59863	12,77468			F1N169	Filamin A
13,52025	16,98103			A7YWG4	Gamma-glutamyl hydrolase
13,46582	19,38284	13,65542		F1MQJ0	Angiotensin-converting enzyme
13,39205	17,9769	17,16153		F1MBC5	Coagulation factor IX
13,37843	15,57288	12,73344		E1BMX5	Neuropilin
13,34068	17,1322			F1MQC0	Gastric inhibitory polypeptide
13,288	17,78972	11,50958		Q9GLX9	Spondin-1
13,22412	20,62132	15,78964		A2VDQ6	Cadherin 11
13,17485	11,52302	9,25899		F1N757	Titin
13,15968	17,64737			F1MYX5	Lymphocyte cytosolic protein 1
13,08875	17,88858	15,20537		F1MPE1	CD109 molecule
13,07486	17,15551	13,45789		Q2KJH9	4-trimethylaminobutyraldehyde dehydrogenase
13,06387	19,29071	15,20751		P19120	Heat shock cognate 71 kDa protein
12,60212	12,6154			F1MM03	Neural cell adhesion molecule 2
12,59481	16,0157	10,33002		E1BIG6	Transferrin receptor
12,13971	19,22303	12,75624		H7BWW2	Beta-hexosaminidase
11,90234	13,86341			F1MNL4	Neogenin 1
11,40344	17,66331	14,50662		Q32LP0	Fermitin family homolog 3

11,31317	14,83832	10,7543		G5E5W1	Coagulation factor VIII
	13,7628	14,30021		F1MVK1	Uncharacterized protein
	18,8662	16,4606		P60712	Actin, cytoplasmic 1
	22,72057	14,68327		E1BMJ0	Factor XIIa inhibitor precursor
	19,24428	14,83644		F1MPD1	Mannose receptor C type 2
	19,67444	13,77726		Q29437	Primary amine oxidase, liver isozyme
	22,61293	16,54102		A6QP30	CPN2 protein
	13,49897	11,20408		F1MC45	Complement factor H precursor
	16,68606	13,0516		F1N0R5	von Willebrand factor
	17,78008			E1BAU4	Protocadherin 17
	19,54263	16,18464		Q3MHL4	Adenosylhomocysteinase
		16,24262		E1BEL6	Neuropilin
	19,67847	10,69784		E1BL29	Bleomycin hydrolase
	20,09365	18,16384		Q3SZZ9	FGG protein
	18,60128			P79136	F-actin-capping protein subunit beta
	18,46925	9,681063		Q29RU2	Oncoprotein-induced transcript 3 protein
	19,26911	15,039		A0JN47	Plexin domain containing 2
	17,78737			A5D9E9	Complement C1r subcomponent precursor
	17,73516	13,48445		Q2KJH4	WD repeat-containing protein 1
	19,18323	13,07561		Q0VD19	Sphingomyelin phosphodiesterase
	17,53001	13,13944		A5D7F4	PCDHGC3 protein
	16,79741			A6H7H6	CDH17 protein
	16,80637	11,06191		E1BB36	Mannosyl (alpha-1,6-)-glycoprotein beta-1,2-N-acetylglucosaminyltransferase
	17,5173	16,02371		F1MVS9	Mannan binding lectin serine peptidase 1
	16,45346	14,41277		Q0VCM4	Glycogen phosphorylase, liver form
	17,99744	11,24079		Q58CQ2	Actin-related protein 2/3 complex subunit 1B
	17,03176	11,44734		Q5EAD2	D-3-phosphoglycerate dehydrogenase
	18,11324	11,31243		A4IFL5	Fibroblast growth factor receptor
	16,67838	13,46531		G1K1R6	Galactokinase
	13,45481	18,4578		A6QL81	DKK3 protein
	16,33872	10,67842		F1MJ04	Protocadherin-18
	12,61549			E1BNR0	Apolipoprotein B
	19,43312			F1MKS3	Thioredoxin domain-containing protein 5 precursor
	16,75551	13,8316		F1N1W7	Neural cell adhesion molecule 1
	17,91876			Q08E66	WAP, Kazal, immunoglobulin,

					Kunitz and NTR domain-containing protein 2
	18,77398			F1MRZ8	Pleckstrin
	16,35459	13,50668		Q3SZJ0	Argininosuccinate lyase
	17,94627			F1MTZ1	Protein NDRG2
	16,65858	13,58344		F1MX49	Platelet-derived growth factor receptor alpha
	17,26316	15,75708		Q3SWX5	Cadherin-6
	18,18863	13,2068		Q9TTJ5	Regucalcin
	18,36956			A5PK77	SERPINA11 protein
	17,61655			F1MJM4	Biotinidase
	14,66445			A7E3Q8	Plastin-3
	17,52145	12,80215		F1MRG7	Actin-related protein 2
	16,9187	14,45507		P68252	14-3-3 protein gamma
	16,58901	12,57792		Q9XSJ4	Alpha-enolase
	14,98175			P52176	Matrix metalloproteinase-9
	13,60837			F1MME1	Uncharacterized protein
	16,97656	11,19488		F1MTJ5	Olfactomedin 2
	15,24719			P33545	Desmocollin-2 (Fragment)
	16,60405	12,55921		F1N468	Uncharacterized protein
	14,1953	10,49286		F1N7T2	Alpha-mannosidase
	16,02933	11,87298		G3N0I4	Cytosol aminopeptidase
	18,34464	17,42021		G3X6Y4	Osteomodulin
		14,92741		Q76LV1	Heat shock protein HSP 90-beta
	19,67687			Q3SYR5	Apolipoprotein C-IV
	16,29887			Q5E9I4	MGAT1 protein
	15,12658			Q5EA01	Beta-1,4-glucuronyltransferase 1
	16,76979			Q5EA20	4-hydroxyphenylpyruvate dioxygenase
	18,43982			Q6UC88	Lymphatic vessel endothelial hyaluronic acid receptor 1
	15,20109			Q58CQ9	Pantetheinase
	14,08032			O77783	Exostosin-2
	14,04388			A7E3W4	Transketolase
	12,51086	8,327598		A7MBJ4	Receptor-type tyrosine-protein phosphatase F
	16,48815			E1B9K8	Sulfotransferase
	15,06575			F1MUP9	Vesicle amine transport 1
	19,22557	15,07096		F1N4I2	Signal transducing adaptor molecule 2
		16,02074		P62157	Calmodulin
	16,38665			P00744	Vitamin K-dependent protein Z
	16,50566			G3X8D7	Glutathione peroxidase
	21,47546			P12820	Angiotensin-converting enzyme (Fragment)
	15,12412			Q1JP79	Actin-related protein 2/3 complex

					subunit 1A
	15,05236			Q2KIV9	Complement C1q subcomponent subunit B
	16,01992			Q2KIW1	Paraoxonase 1
	16,65102	11,4919		Q3MHR7	Actin-related protein 2/3 complex subunit 2
	15,8739			Q3SZ79	Hepatic triacylglycerol lipase
	16,66886			Q3T035	Actin-related protein 2/3 complex subunit 3
	15,90485			Q3T0E5	Adipocyte plasma membrane-associated protein
	15,56938			Q3T0P6	Phosphoglycerate kinase 1
	13,66233	10,36446		Q3ZBV8	Threonine--tRNA ligase, cytoplasmic
	15,89707			Q5E9H0	Phospholipase A1 member A
